# Red Squirrel Middens Influence Abundance but Not Diversity of Other Vertebrates

**DOI:** 10.1371/journal.pone.0123633

**Published:** 2015-04-29

**Authors:** Erin E. Posthumus, John L. Koprowski, Robert J. Steidl

**Affiliations:** Wildlife and Fisheries Science, School of Natural Resources and the Environment, University of Arizona, Tucson, Arizona, United States of America; University of South Carolina, UNITED STATES

## Abstract

Some animals modify the environment in ways that can influence the resources available to other species. Because red squirrels (*Tamiasciurus hudsonicus*) create large piles of conifer-cone debris (middens) in which they store cones, squirrels concentrate resources that might affect biodiversity locally. To determine whether other animals are attracted to midden sites beyond their affinity for the same resources that attract red squirrels, we assessed associations between middens, mammals, and birds at population and community levels. We surveyed 75 middens where residency rates of red squirrels varied during the previous five years; sampling along this residency gradient permitted us to evaluate the influence of resources at middens beyond the influence of a resident squirrel. At each location, we quantified vegetation, landscape structure, abundance of conifer cones, and midden structure, and used capture–recapture, distance sampling, and remote cameras to quantify presence, abundance, and species richness of mammals and birds. Red squirrels and the resources they concentrated at middens influenced mammals and birds at the population scale and to a lesser extent at the community scale. At middens with higher residency rates of red squirrels, richness of medium and large mammals increased markedly and species richness of birds increased slightly. After accounting for local forest characteristics, however, only species richness of medium-to-large mammals was associated with a red squirrel being resident during surveys. In areas where red squirrels were resident during surveys or in areas with greater amounts of resources concentrated by red squirrels, abundances of two of four small mammal species and two of four bird species increased. We conclude that the presence of this ecosystem modifier and the resources it concentrates influence abundance of some mammals and birds, which may have implications for maintaining biodiversity across the wide geographic range inhabited by red squirrels and other larderhoarding animals.

## Introduction

Certain species influence ecosystems disproportionally [1–2] or perform unique ecological functions [3]. If extirpated, the loss of these species could influence species diversity and ecosystem function adversely [4], earning them the moniker of keystone species [5]. Although the utility of the keystone-species concept has been debated, evaluating the interaction strength of particular species can increase our understanding of factors that govern ecological processes [1, 6].

A suite of mammals that vary widely both taxonomically and functionally have been hypothesized to function in these ecologically important ways, including beaver (*Castor canadensis*) [7], prairie dogs (*Cynomys* spp.) [8], kangaroo rats (*Dipodomys* spp.) [9], sea otters (*Enhydra lutris*) [10], and bison (*Bison bison*) [11]. Species that redistribute resources or create structures on a large scale have been further classified as keystone modifiers [6] or ecosystem engineers [12]. Examples include beaver, which alter hydrology and productivity by building dams and through their feeding activities [7], prairie dogs, which alter soil structure and vegetation composition through burrowing and feeding [8], badgers (*Taxidea taxus*), which create mounds that maintain diversity of prairie flora [13], and woodrats (*Neotoma* spp.), which alter rates of nitrogen mineralization in soils and that build nests with a unique microclimate used by other animals [14].

**Fig 1:**
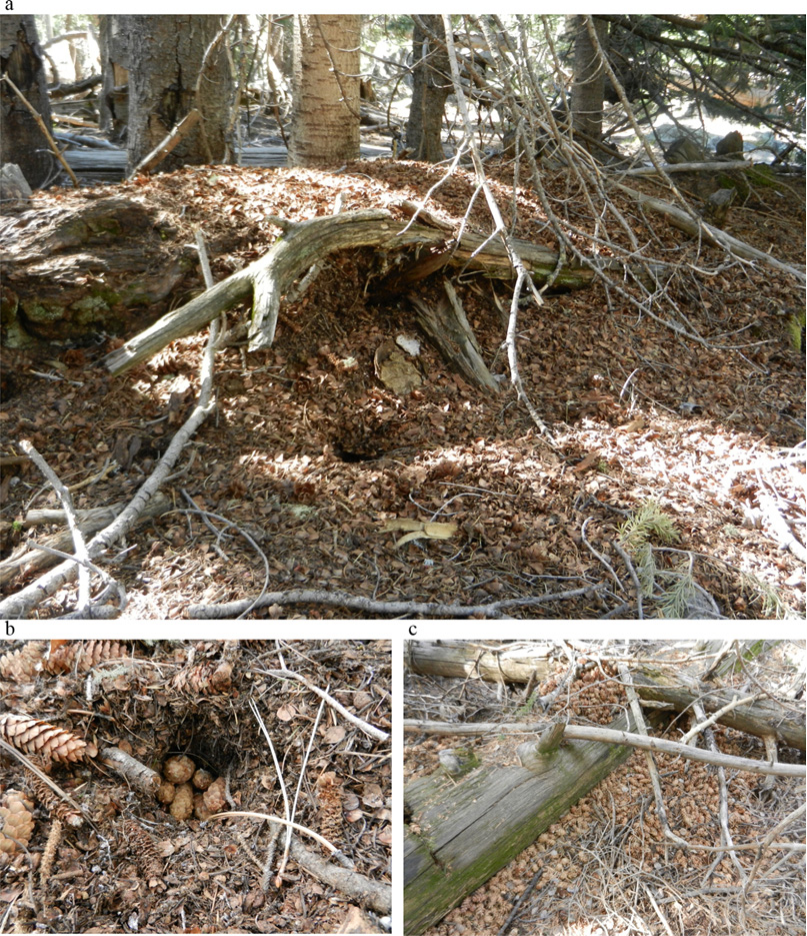
Red squirrel (*Tamiasciurus hudsonicus*) midden. Photographs of (a) red squirrel (*Tamiasciurus hudsonicus*) midden cone-scale pile, (b) cached cones inside pit excavated by red squirrel, and (c) stored cones, which may number in the thousands at a single midden. Photo credit, E. E. Posthumus. Mt. Graham, Graham Co. Arizona, 2011–2012.

Some species also alter the distribution of resources, concentrating resources for themselves and in ways that could increase the availability of resources for other species [15]. Handling and storage of food by animals for later use, termed larderhoarding, enables animals to satisfy energy requirements when resource abundance fluctuates [15]. Red squirrels (*Tamiasciurus hudsonicus*), a territorial tree squirrel distributed throughout most coniferous forests of the United States and Canada [16], often larderhoard food supplies that last for years [15, 17]. Red squirrels fulfill a number of important ecosystem functions, including dispersing seeds, serving as prey for predators, and creating structure via conspicuous cone-scale piles, known as middens, which are a product of feeding in a single location (Fig 1) [16]. Middens are central to a single red squirrel’s well-defended territory [16], which can vary from 1 to >10 ha [18]. In the southwestern United States, middens are typically located in forest patches with locally dense canopies, high stem densities, thick foliage, and on cooler north-facing slopes [19–20], and have a cool, moist microclimate optimal to larderhoard conifer cones and fungi [21–22]. Middens often are used over multiple generations [17] and can reach 13 m in diameter and 50 cm in depth [23]. The structure of middens facilitates tunneling, nesting, and access to thousands of stored cones, plus seeds dropped during feeding are distributed throughout a red squirrel’s territory [24]. These resources may attract small mammals and birds, which may in turn attract other predatory mammals and birds [25–26].

Positive associations with middens have been reported for marten (*Martes americana*) that use subnivean tunnels more often when they are near middens [27], and grizzly bears (*Ursus arctos horribilis*) that excavate middens to obtain seeds of whitebark pine (*Pinus albicaulis*) [28]. Richness and abundance of mammals and birds has been observed to be higher at middens than in surrounding forest [25–26].

Although species richness of vertebrates may be higher at middens, this might be an artifact of species selecting forest features that are coincident with those used by red squirrels [26] rather than species leveraging resources concentrated by red squirrels. Red squirrels select sites with high canopy cover, large cone-bearing trees [19–20], and coarse woody debris [26], features likely important to many other species. For example, deer mice (*Peromyscus* spp.), chipmunks (*Tamias* spp.), voles (*Microtus* spp.), and woodrats consume conifer seeds [29] and den in coarse woody debris [30–31]. Insectivores, such as shrews (Soricidae), may be attracted to the abundant insects supported by conifer seeds and in moist conditions; flying squirrels (*Glaucomys* spp.) and voles may target woody debris that is conducive to growth of fungi [32].

To determine whether animals are attracted to middens beyond their affinity for resources similar to those selected by red squirrels, we assessed associations between middens, mammals, and birds at population and community scales across a gradient of residency rates by squirrels during the previous five years. Our objective was to determine if red squirrels or the resources they concentrate influence populations and communities of vertebrates. If the presence of middens influences other species, we anticipate that richness and abundance of mammals and birds will be higher at locations with higher residency rates and will be associated positively with the presence of red squirrels at middens and abundance of the resources they concentrate.

## Materials and Methods

Field efforts were conducted under permits from the United States Department of Agriculture Forest Service, Arizona Game and Fish Department, and United States Fish and Wildlife Service, and approved by the University of Arizona Institutional Animal Care and Use Committee (IACUC; Protocol #11–248). We studied a 100-ha area of mixed-conifer forest at an elevation of 2,870 to 3,050 m in the Pinaleño Mountains, 25 km southwest of Safford, Graham Co., Arizona. The forest is dominated by Douglas-fir (*Pseudotsuga menziesii*), white fir (*Abies concolor*), and southwestern white pine (*Pinus strobiformis*), interspersed with cork-bark fir (*Abies lasiocarpa* var. *arizonica*), Engelmann spruce (*Picea engelmanii*), aspen (*Populus tremuloides*) and ponderosa pine (*Pinus ponderosa*) [33]. Middens in this area have been surveyed for red squirrels every three months since 1996 [33] because endemic Mt. Graham red squirrels (red squirrel; *T*. *h*. *grahamensis*) are listed as endangered in the United States.

In 2011, we classified 176 locations based on residency rates of red squirrels during the previous five years, which we defined as the proportion of quarterly surveys where a red squirrel was observed at each midden. Surveys involved visiting middens to determine if each was inhabited based on signs of red squirrel activity (tracks, feeding sign, nesting material) [33]. Because red squirrels are highly territorial and solitary, when middens are inhabited, they are usually defended aggressively by the resident adult [33]. From this set of midden locations, we selected 50 in both 2011 and 2012, 10 at random from each of five residency rate classes to capture the entire gradient: 0% residency since 1996, 0% residency since 2011, 1–49% and without a resident red squirrel for at least one year prior to the study, 50–75% residency, and > 75% residency. Fifteen locations were included in both 2011 and 2012 samples, which we treated as independent across years. Distance between locations averaged 75 m (min = 30 m, max = 150 m).

### Habitat Features

We surveyed vegetation along 2-m wide and 30-m long transects that radiated from the center of each location in four cardinal directions (total area surveyed = 0.02 ha/location). We used a spherical densiometer to estimate canopy closure [34] and averaged readings taken 0, 2, 10, 20, and 30 m from center of each location in each direction. We counted the number of live and dead trees in three size classes (<20 cm, 20–40 cm, >40 cm dbh), measured diameter at breast height (dbh), and calculated basal area/ha for all live and dead trees. We recorded the number of live trees of each species and calculated diversity at each location with the Shannon-Weiner index. We measured volume of hard downed logs >20 cm in diameter and slope and aspect in absolute degrees from the center of each location.

### Resources Concentrated by Red Squirrels

We recorded whether a red squirrel was resident at each location between May and September of each year (red squirrel residency during surveys), and measured area and depth of cone-scale piles at each midden. We ranked the number of cones cached in each scale pile from 1–4 to reflect 1–25, 26–50, 51–75, and >75 cones, respectively, and used the highest value observed in either September or December of the previous year to approximate cone availability.

### Composition of Mammal and Bird Communities

We surveyed small mammals at all locations using a trapping-web design centered on each location [35]. Webs were comprised of eight, 30-m lines radiating at 45° angles from the center of the location. Along each line, we set one folding, galvanized Sherman live trap (7.5 by 9 by 23 cm: Model LFG, H.B. Sherman Trap Co., Tallahassee, FL) at 10, 20, and 30 m from the center of each location, four traps at 2 m in each cardinal direction, and one trap at the center for a total of 29 traps per location. For one four-night period at each location per year between May and September, we baited traps at sunset with a mix of peanut butter, rolled oats, and alfalfa pellets, checked traps at sunrise, and reset and checked again mid-morning. We identified individuals captured to species, and weighed, measured [36], and marked each individual with ear tags (Monel #1005–1, National Band and Tag Company, Newport, KY), before releasing animals at the capture location. Handling methods followed American Society of Mammalogists [37] and University of Arizona IACUC guidelines.

We surveyed medium and large mammals at all locations with remote cameras (Bushnell Trophy Cam 119436c, TrailCamPro, Springfield, MO) [25]. We set three cameras 5–10 m from and facing the center of each survey location and located 1–2 m above the ground for a six-day period in each year established at random between May and September. We set cameras at maximum sensitivity to record one photo upon detection, with a 3-sec delay between photographs. We identified animals in photographs to species and counted the number of species detected over the six-day period for each location.

We surveyed birds from each location with 10-min point counts on four consecutive mornings (0500–0900 h) concurrent with small mammal trapping [38]. We detected birds by sight and sound and classified distances from the observer as 0–10 m, 11–20 m, 21–30 m, 31–50 m, and 51–100 m, truncating detections at 100 m to include only those birds proximate to middens. We considered only species that were ground foragers to target species that search for seeds or insects on the ground [39].

### Data Analysis

We used logistic regression for binomial counts (number of quarterly surveys where a red squirrel was present relative to the total number of quarterly surveys done at each site) to explore relationships between residency rates and habitat features. We used linear regression to explore relationships between residency rates and species richness of small mammals, medium and large mammals, and birds, and added quadratic and cubic components successively until they were no longer significant (*P* > 0.10) when relationships were curvilinear [40]. To reduce dimensionality among the seven habitat features we measured, we used principal components analysis based on the correlation matrix after first transforming basal area, number of large trees > 40 cm dbh, number of large snags > 40 cm dbh, volume of downed logs, and slope with the natural log and by squaring canopy cover to normalize their distributions. To test whether species richness was associated with a red squirrel being resident during surveys or the resources concentrated by red squirrels after accounting for forest characteristics, we first used Poisson regression to model richness of small mammals, medium and large mammals combined, and birds separately as a function of habitat features as described by the first three principal components. We retained influential components (*P* < 0.10), then fit a second model to describe the influence of red squirrels that included red squirrel residency during surveys, volume of the cone-scale pile, and index of cached cones. We then used a drop-in-deviance test to contrast the amount of variation in the response explained by the two models and to determine the influence of red squirrels on each response after accounting for habitat features [41].

For all mammal and bird species observed, we used logistic regression to model presence of each species at each location and as a function of residency rates of red squirrels. For the two mammal species captured at > 90% of sites, deer mice (*Peromyscus maniculatus*) and cliff chipmunks (*Tamias dorsalis*), we created hierarchical models of abundance based on a capture-recapture framework with the R package ‘unmarked’ [42]. To model the detection process, we assumed a behavioral response to trapping (M_b_, based on our preliminary modeling) and explored the influence of mean low daily temperature and mean daily precipitation for each four-day trapping period (National Wildfire Coordinating Group 2012, from a weather station within our study area) and retained influential covariates (*P* < 0.10). To determine whether abundance of *P*. *maniculatus* and *T*. *dorsalis* was associated with a red squirrel being resident during surveys or the resources concentrated by red squirrels after accounting for habitat features, we fit two models using a process similar to the one we described for species richness.

For all ground-foraging bird species, including American robin (*Turdus migratorius*), hermit thrush (*Catharus guttatus*), Stellar’s jay (*Cyanocitta stelleri*), and yellow-eyed junco (*Junco phaeonotus*), we created hierarchical models for abundance based on a distance-sampling framework with ‘unmarked’ [42]. We compared half normal, hazard rate, and uniform detection functions, explored a series of models for detection probability including models that varied with behavior and mean low temperature and precipitation, and retained influential covariates (*P* < 0.10). To determine whether abundance of each species was associated with a red squirrel being resident during surveys or the resources concentrated by red squirrels after accounting for vegetation features, we used a process similar to the one we described for species richness.

## Results

### Habitat Features Surrounding Middens

**Fig 2:**
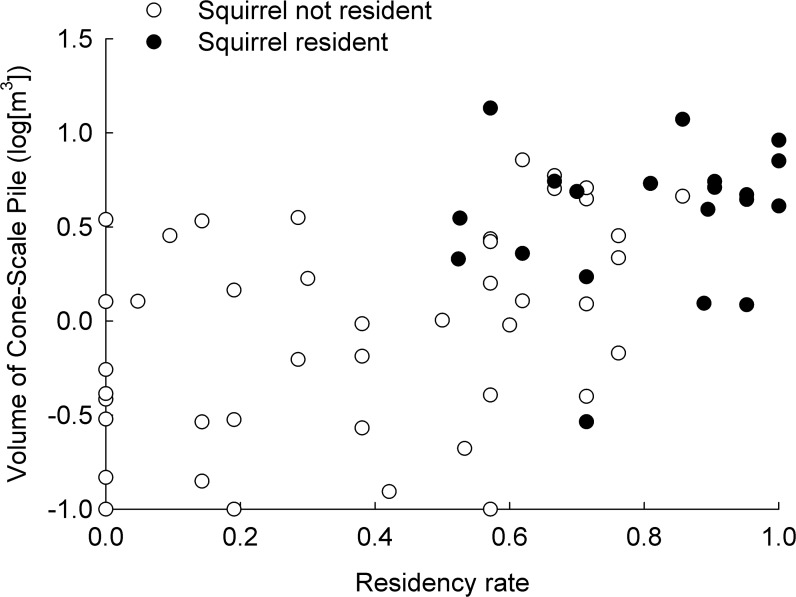
Relationship between red squirrel residency rates and resources concentrated by red squirrels. Relationship between resources concentrated by red squirrels, red squirrel residency during surveys, and residency rate (proportion of quarterly surveys during the previous five years) of red squirrels at middens, Mt. Graham, Graham Co. Arizona, 2011–2012.

Residency rates of red squirrels increased with canopy cover (7.16 ± 0.57% [SE], χ^2^ = 157.02, *P* < 0.001), basal area (15.57 ± 1.36 m²/ha, χ^2^ = 79.63, *P* < 0.001), number of large trees > 40 cm dbh (1.78 ± 1.11 trees/ha, χ^2^ = 33.02, *P* < 0.001), number of large snags > 40 cm dbh (1.31 ± 1.07 snags/ha, χ^2^ = 16.62, *P* < 0.001), steeper slopes (2.65 ± 1.20°, χ^2^ = 28.50, *P* < 0.001), more north facing slopes (-0.0016 ± 0.0010°, χ^2^ = 2.88, *P* = 0.090), and decreased with the index of tree diversity (-0.53 ± 0.16, χ^2^ = 10.70, *P* < 0.001) and volume of downed logs (-2.08 ± 1.19 m^3^, χ^2^ = 17.34, *P* < 0.001). Volume of cone-scale piles (4.75 ± 0.55 m^3^, *t*_99_ = 8.64, *P* < 0.001) and the index of number of cached cones (2.55 ± 0.30, *t*_99_ = 8.47, *P* < 0.001) increased as residency rates increased, indicating that resources concentrated by red squirrels varied with residency rates (Fig 2).

Most (60.7%) of the variation in the eight original habitat features was captured by the first three principal components. The first principal component (25.4% of variation) described aspects of forest density, with high loadings on percent canopy cover (factor weight = 0.81), north facing slopes (0.72), number of live trees >40 cm dbh (0.62), and low tree diversity (0.51). The second principal component (19.4%) described aspects of tree structure, with high loadings on number of large snags >40 cm dbh (0.83) and basal area (0.66). Lastly, the third principal component (15.8%) described aspects of the forest floor, including volume of downed logs (0.89) and flatness of slopes (0.58). Residency rates were associated with the first (*r* = 0.257, *P* = 0.010), second (*r* = 0.195, *P* = 0.053) and third principal component (*r* = 0.192, *P* = 0.055).

### Influence of Resources Concentrated by Red Squirrels on Vertebrates

We captured six species of small mammals during 11,600 trap nights (Table 1). Species richness of small mammals was not associated with residency rate of red squirrels (*t*_99_ = 0.94, *P* = 0.350, *r*² = 0.009). After accounting for habitat features, species richness of small mammals was not influenced by a red squirrel being resident during surveys or resources concentrated by red squirrels (Tables 2 and 3). We observed 8 species of medium and large mammals in 1722 camera days in addition to red squirrels (Table 1). Species richness of medium and large mammals increased as residency rates of red squirrels increased (0.98 ± 0.32 species, *t*_99_ = 3.34, *P* = 0.001, *r*² = 0.102). After accounting for forest characteristics, species richness of medium and large mammals increased when a red squirrel was resident during surveys (Tables 2 and 3). We detected 25 species of birds, including four that we classified as ground foragers (Table 1). Species richness of birds increased slightly as residency rate of red squirrels increased (1.29 ± 0.77 species, *t*_99_ = 1.65, *P* = 0.101, *r*² = 0.027). After accounting for habitat features, species richness of birds was not influenced by a red squirrel being resident during surveys or resources concentrated by red squirrels (Table 2), but did increase with forest density (Table 3).

**Table 1:** Proportion of sites surveyed at which mammals and birds were observed.

		Proportion of sites	Residency	
Group	Species		*χ* ^*2*^	*P*
Small mammals	*Microtus longicaudus*	0.32	0.01	0.929
	*Neotoma mexicana*	0.59	0.60	0.439
	*Peromyscus boylii*	0.07	1.82	0.178
	*Peromyscus maniculatus*	1.00		
	*Sorex monticolus*	0.40	1.59	0.207
	*Tamias dorsalis*	0.94	1.75	0.186
Medium and large mammals	*Lynx rufus*	0.12	5.61	0.018
	*Mephitis mephitis*	0.26	0.02	0.903
	*Odocoileus virginianus*	0.09	1.62	0.202
	*Otospermophilus variegatus*	0.53	0.01	0.926
	*Puma concolor*	0.02	0.44	0.506
	*Sciurus aberti*	0.09	0.06	0.802
	*Urocyon cinereoargenteus*	0.04	1.37	0.241
	*Ursus americanus*	0.26	4.22	0.040
Ground-foraging birds	*Catharus guttatus*	0.74	0.00	0.989
	*Cyanocitta stelleri*	0.19	2.73	0.098
	*Junco phaeonotus*	0.86	0.46	0.498
	*Turdus migratorius*	0.34	0.15	0.695

Chi-square and *P*-values from logistic regression models comparing proportion of sites surveyed at which mammal and bird species were observed across the gradient of residency rates for red squirrels (*n* = 100).

**Table 2:** Estimates ± standard errors and test statistics with associated *P*-values from models of species richness and abundance contrasting sites where red squirrel were and were not resident during surveys.

		Red squirrel			
Metric	Group	Resident	Not Resident	*χ* ^*2*^	*P*
Richness	Small mammals	3.40 ± 0.17	3.28 ± 0.12	0.38	0.944
	Medium and large mammals	2.60 ± 0.26	1.35 ± 0.11	14.96	0.002
	Birds	7.90 ± 0.67	6.78 ± 0.30	2.52	0.471
Abundance	*Peromyscus maniculatus*	17.32 ± 4.11	24.8 ± 5.36	11.43	0.010
	*Tamias dorsalis*	7.02 ± 1.78	14.68 ± 1.78	18.79	< 0.001
	*Catharus guttatus*	4.63 ± 1.04	2.39 ± 0.33	8.45	0.038
	*Cyanocitta stelleri*	0.30 ± 0.20	0.40 ± 0.14	1.43	0.698
	*Junco phaeonotus*	49.05 ± 9.99	55.98 ± 5.68	3.31	0.346
	*Turdus migratorius*	4.34 ± 1.58	1.32 ± 0.29	10.13	0.017

Species richness of three taxonomic groups and abundance of two small mammal and four bird species in areas with and without a red squirrel resident during surveys. Chi-squared statistics and *P*-value (all with degrees of freedom = 3) are from drop-in-deviance tests comparing models with and without a resident red squirrel.

**Table 3:** Parameter estimates, standard errors, and *P*-values from models assessing the importance of a red squirrel being resident and resources concentrated by red squirrels during surveys to species richness of mammals and birds.

Group	Parameter	Estimate	*SE*	*P*
Small mammals	Intercept	1.17	0.07	< 0.001
	Squirrel resident during surveys	-0.01	0.19	0.952
	Volume of cone-scale pile (m^3^)	< 0.01	0.03	0.951
	Index of cached cones	0.02	0.05	0.286
Medium and large mammals	Intercept	0.26	0.10	0.013
	Squirrel resident during surveys	0.49	0.34	0.043
	Volume of cone-scale pile (m^3^)	0.03	0.03	0.330
	Index of cached cones	0.02	0.06	0.797
Birds	Intercept	1.92	0.05	< 0.001
	Forest density	0.14	0.04	< 0.001
	Tree structure	0.05	0.05	0.164
	Squirrel resident during surveys	0.12	0.12	0.324
	Volume of cone-scale pile (m^3^)	-0.02	0.02	0.300
	Index of cached cones	0.03	0.03	0.428

Variables without units are either indicator variables or were standardized for analysis.

After accounting for forest characteristics, abundance of *P*. *maniculatus* was lower in locations with a red squirrel resident during surveys (-0.36 ± 0.12 individuals) and increased as the number of cached cones increased (0.08 ± 0.03 individuals/cone index unit; Tables 2 and 4). Detection probability of *P*. *maniculatus* changed in response to initial capture and with precipitation (Table 4). After accounting for forest characteristics, abundance of *T*. *dorsalis* was lower in locations with a red squirrel resident during surveys (-0.74 ± 0.17 individuals), and increased as the number of cached cones increased (0.11 ± 0.04 individuals/cone-index unit; Tables 2 and 4). Detection probability of *T*. *dorsalis* changed with precipitation (Table 4).

**Table 4:** Parameter estimates from models assessing the importance of resources concentrated by red squirrels and a red squirrel being resident during surveys to abundance of mammals and birds.

Group	Process	Parameter	Estimate	*SE*	*P*
*Peromyscus maniculatus*	Abundance	Intercept	3.14	0.22	< 0.001
		Tree structure	0.06	0.04	0.120
		Forest floor	0.16	0.04	< 0.001
		Squirrel resident during surveys	-0.36	0.12	0.002
		Volume of cone-scale pile (m^3^)	0.01	0.02	0.546
		Cached cone index	0.08	0.03	0.011
	Detection	Initial capture probability	-2.07	0.29	< 0.001
		Recapture probability	-1.31	0.07	< 0.001
		Precipitation (mm)	-17.24	7.21	0.017
*Tamias dorsalis*	Abundance	Intercept	2.37	0.17	< 0.001
		Forest density	0.17	0.06	0.006
		Midden	0.23	0.16	0.144
		Squirrel resident during surveys	-0.74	0.17	< 0.001
		Volume of cone-scale pile (m^3^)	0.04	0.03	0.137
		Cached cone index	0.11	0.04	0.013
	Detection	Intercept	-2.46	0.14	< 0.001
		Precipitation (mm)	0.90	0.36	0.014
*Catharus guttatus*	Abundance	Intercept	0.87	0.14	< 0.001
		Forest density	0.17	0.09	0.052
		Squirrel resident during surveys	0.66	0.26	0.010
		Volume of cone-scale pile (m^3^)	-0.12	0.11	0.257
		Index of cached cones	0.00	0.07	0.996
	Detection	Intercept	4.21	0.65	< 0.001
		Scale	2.00	6.99	0.775
*Cyanocitta stelleri*	Abundance	Intercept	-0.97	0.37	0.009
		Tree structure	0.81	0.30	0.007
		Squirrel resident during surveys	-0.28	0.70	0.689
		Volume of cone-scale pile (m^3^)	0.27	0.32	0.385
		Index of cached cones	0.06	0.19	0.749
	Detection	Intercept	4.08	0.35	< 0.001
		Low temperature	-0.10	0.08	0.196
		Scale	3.90	26.4	0.882
*Junco phaeonotus*	Abundance	Intercept	4.06	0.10	0.000
		Squirrel resident during surveys	-0.13	0.22	0.555
		Volume of cone-scale pile (m^3^)	-0.02	0.08	0.853
		Index of cached cones	-0.05	0.06	0.405
	Detection	Intercept	2.72	0.03	0.000
*Turdus migratorius*	Abundance	Intercept	0.36	0.21	0.081
		Squirrel resident during surveys	1.19	0.47	0.011
		Volume of cone-scale pile (m^3^)	-0.48	0.19	0.011
		Index of cached cones	-0.12	0.13	0.341
	Detection	Intercept	3.99	0.10	0.000
		Precipitation (mm)	-3.78	1.57	0.016
		Scale	2.10	0.64	0.001

Parameter estimates and standard errors for models of abundance of mammals observed at > 90% of sites and ground foraging birds. Variables without units are either indicator variables or were standardized for analysis.

After accounting for habitat features, abundance of *C*. *guttatus* was higher in locations with a red squirrel resident during surveys (0.66 ± 0.26 individuals; Tables 2 and 4). Abundances of *C*. *stelleri* and *J*. *phaeonotus* were not influenced by a red squirrel being resident during surveys or resources concentrated by red squirrels (Tables 2 and 4). Abundance of *T*. *migratorius* was higher in locations where a red squirrel was resident during survey (1.19 ± 0.47 individuals/cone-index unit) and lower at locations with larger cone scale piles (-0.48 ± 0.19 m^3^; Tables 2 and 4). Detection probability of *C*. *stelleri* was influenced by low temperature and detection of *T*. *migratorius* was influenced by precipitation (Table 4). For *C*. *guttatus*, *C*. *stelleri*, and *T*. *migratorius*, a hazard-rate detection function fit best, whereas for *J*. *phaeonotus*, a half-normal detection function fit best.

## Discussion

### Effects of red squirrels on vertebrate communities

Although species classified as keystone modifiers often are thought to explain changes in community-scale attributes, such as species diversity [43], we found that the largest effects of red squirrels on vertebrates was at the population scale. Importantly, changes in species diversity attributed to keystone species are rarely disentangled from overlapping habitat preferences shared by other species. After accounting for the influence of vegetation and landscape characteristics, a red squirrel being resident during surveys and resources concentrated by red squirrels explained little of the variation in species richness for small mammals and birds. However, we observed an increase in species richness of medium and large mammals, principally mesocarnivores and predators, as residency rates of red squirrel middens increased, suggesting heightened activity of animals at red squirrel middens. After removing the effects of vegetation and landscape characteristics, species richness of medium and large mammals increased at locations where a red squirrel was resident during survey. Bobcats may be attracted to middens due to increased prey [44] and black bears excavate middens in search of cones [45]. Mammals may also use middens for thermoregulatory properties, as marten use middens as resting sites in winter [46].

Species richness of birds varied with local habitat features, but was not influenced positively by a red squirrel being resident during surveys or resources concentrated by red squirrel. Middens with high residency rates had high canopy cover and basal area, and squirrels likely select and persist at these sites for protection from avian predators [44], presence of large amounts of cone-bearing trees [47], and moderate temperatures with higher humidity that aid thermoregulation [17] and cone storage [24]. Similarly, areas where the forest canopy is closed likely provide protection from predators, a moderate microclimate, and nesting locations for a variety of species [48–49]. However, as documented in our photographs, avian ground foragers used the surface of the cone-scale pile at locations where a red squirrel was resident during surveys, perhaps to forage for seeds or insects [39]. We recommend further study on the abundance and diversity of arthropods in cone-scale piles to understand whether middens are a hotspot for arthropod diversity as found at structures created by other keystone modifiers such as packrats and kangaroo rats [9,14].

### Effects of red squirrels on species abundances

Resources concentrated by red squirrels had their largest effects at the population scale for a number of common small mammals and birds. We detected *P*. *maniculatus* at all locations and *T*. *dorsalis* at all but six locations. These species are generalists, and their diet may include seeds of conifers, shrubs, grasses and forbs as well as insects, snails, fungi, bones, leaves or bark [29]. Abundances of *P*. *maniculatus* and *T*. *dorsalis* were associated positively with food resources at middens. *P*. *maniculatus* live and nest in logs and under debris piles [29], which may result in an association of *P*. *maniculatus* with the food resources of the midden cone-scale pile. This is supported by photographs of these species on the cone-scale pile, including images of *T*. *dorsalis* holding conifer cones that may have been taken from the midden. *T*. *dorsalis* also tunnels and creates dens and nests in many substrates [50], which may be facilitated by the microclimate and structure of the cone-scale pile.

Abundances of *T*. *migratorius* and *C*. *guttatus* were associated positively with a red squirrel being resident during surveys. Ground-foraging birds were the only birds that we detected with cameras at the cone-scale pile. The diet of these birds is composed primarily of seeds and invertebrates [39], which may be higher with a resident red squirrel dropping seeds while feeding [24], and might explain detections of these birds on the surface of the cone-scale pile.

### Conclusions

By producing a midden, red squirrels modify the distribution of resources and influence local forest structure and microclimate in ways that can persist for decades when occupied serially by residents [24, 33]. Although red squirrel middens support higher levels of vertebrate diversity and activity, most of their effect is due to vegetation characteristics associated with sites selected for construction of middens [19, 20]. Middens provide a visible structure that indicates the availability of resources that we found to be associated with increased occurrence and abundance of numerous other species. Knowledge of the important function of middens in influencing patterns in animal diversity, and potentially larderhoards of other species [15], is critical to our understanding of ecosystem function.

Although species richness did not increase directly as a result of red squirrel middens for all taxa, our finding that red squirrels are associated with high levels of mammal and bird diversity due to a common affinity for key forest characteristics is important. Middens serve as conspicuous indicators of diversity in forests. The Mt. Graham red squirrel persists at the southernmost extent of the distribution of red squirrels [29], an area with warmer, drier forests than in much of the rest of the species range. At the southern end of the range, forest disturbances, such as loss of canopy cover from insect infestations or fire, which are likely to become more frequent with climate change [4], threaten to alter midden microclimate to the detriment of food storage [22]. With predicted global change creating warmer, drier climates, and threatening montane diversity [51], such challenges in southern forests may portend the future of forest communities at northern latitudes.

Our results suggest that although red squirrels are associated with increased species richness their primary impact occurs at the population scale for several common species. Abundance can be influenced through multiple pathways, including indirect facilitation as has been observed in kangaroo rats [9, 52]. Although mechanisms for these processes are unknown, the influence of red squirrels on multiple dominant species within the forest suggests a subtle impact with potential links to pathways of influence observed in many ecosystem engineers [6, 53]. Increased knowledge of the interaction strength between other ecosystem modifiers or larderhoarders and their environments may help to inform decisions related to forest management and restoration and offer insight on the conservation value of these species.

## Supporting Information

S1 Dataset Data used in analyses. (XLSX)
